# The Hidden Burden of Cyanotic Nephropathy: Prevalence and Correlates in Indian Children With Cyanotic Congenital Heart Disease at a Tertiary Center

**DOI:** 10.7759/cureus.91883

**Published:** 2025-09-09

**Authors:** Faizul Furquan Ansari, Vinod Kumar, Yash Shrivastava, Anish Gupta, Bela Goyal, Nowneet Bhat

**Affiliations:** 1 Department of Pediatrics, All India Institute of Medical Sciences, Rishikesh, IND; 2 Department of Pediatrics, King George's Medical University, Lucknow, IND; 3 Department of Cardiothoracic and Vascular Surgery, All India Institute of Medical Sciences, Rishikesh, IND; 4 Department of Biochemistry, All India Institute of Medical Sciences, Rishikesh, IND

**Keywords:** albumin-to-creatinine ratio (acr), cyanotic congenital heart disease, cyanotic nephropathy, protein-creatinine ratio (pcr), renal impairment

## Abstract

Background: We observed a paucity of data on the prevalence of nephropathy in cyanotic heart diseases, with limited studies focusing on glomerular and tubular damage due to chronic hypoxia.

Method: Over a period of 18 months, children with cyanotic congenital heart disease (CCHD) in the age range of 2-18 years attending the outpatient department or admitted to the wards were enrolled. Using basic laboratory parameters, including serum creatinine, urine albumin, urine protein, and urine creatinine, we calculated the albumin-to-creatinine ratio (ACR), protein-to-creatinine ratio (PCR), and estimated glomerular filtration rate (eGFR) to identify renal impairment.

Result: Among the study population (n = 98), 80.61% had albuminuria (ACR > 30 mg/g of creatinine), and 54.08% had increased PCR (>0.2 mg/mg of creatinine). Based on eGFR, 39.79% had a GFR of less than 90 mL/minute (n = 39). Two (n = 2) had nephrotic range proteinuria, and none of the patients were in chronic kidney disease stage 4 or 5. Age-wise analysis revealed a higher prevalence of renal impairment in the 11-18-year age group compared to the two-to-five-year age group, indicating progressive renal dysfunction with uncorrected CCHD. Hematocrit (HCT) levels showed a significant association with renal impairment, with higher HCT levels (>65%) correlating with increased prevalence of renal impairment. Hypoxemia and lower oxygen saturation levels were also associated with increased albuminuria, suggesting their contribution to renal damage.

Conclusion: The study highlights the prevalence of latent nephropathy (80.61%) in CCHD patients and its association with HCT levels, emphasizing the need for early recognition and monitoring of proteinuria and renal function.

## Introduction

Congenital cyanotic heart disease (CCHD) is a common form of congenital heart disease. It is associated with cyanotic nephropathy (CN), causing progressive renal function decline [1]. The outcome and prognosis of CN indicate that adult patients with CCHD are prone to develop functional and structural kidney abnormalities [2,3]. CN is characterized by proteinuria, hypertension, and decreased glomerular filtration rate (GFR) [4]. Factors contributing to CN include hypoxia severity, elevated hematocrit (HCT) levels, and chronic hypoxia [5]. Microalbuminuria is a recognized biomarker; however, its application in CN is limited, primarily due to a lack of awareness and the scarcity of studies on this topic.

Microalbuminuria, measured by urinary albumin-to-creatinine ratio (ACR), is an early indicator of glomerular damage and progressive renal disease [6]. According to the Kidney Disease: Improving Global Outcomes 2012 guidelines on management of chronic kidney disease, albuminuria is considered a suitable marker of kidney damage (owing to increased glomerular permeability). Specifically, a urine albumin excretion rate of >30 mg/24 hours is considered significant, which is approximately equivalent to a urine ACR of >30 mg/g of creatinine (the normal urine ACR in young adults is <10 mg/g) [7].

Hongsawong et al. studied 94 children with CCHD and found the prevalence of CN of 92.55% based on significant proteinuria staging and 58.51% based on albuminuria staging, with 31.9% having lower GFR. Higher HCT, platelet count, and longer waiting times correlated with worse outcomes [8].

Early corrective cardiac surgery can prevent renal dysfunction, which can manifest in early childhood and worsen with longer periods of cyanosis and higher levels of erythrocytosis. The duration of cyanosis and higher HCT levels in children with CCHD are closely associated with the risk of renal injury [9,10].

Regular monitoring of microalbuminuria, blood pressure, and GFR using creatinine or cystatin C-based formulas is recommended, alongside angiotensin-converting enzyme (ACE) inhibitors and strict blood pressure control [11]. Timely identification by a pediatric nephrologist is essential for better outcomes.

There is a lack of studies in the Indian setup estimating the prevalence of nephropathy associated with congenital heart diseases. CN is a significant complication, making research on its prevalence highly relevant.

## Materials and methods

Study setting and subjects

This is a prospective cross-sectional study, which was carried out at the Department of Pediatrics, All India Institute of Medical Sciences, Rishikesh, over a period of 18 months from November 2020 to April 2021. This study is designed to calculate the prevalence of CN in children with CCHD. For the conduct of the study, approval from the Institute Ethical Committee (IEC) was obtained with the IEC number “AIIMS/IEC/21/50” dated 12/02/2021.

The study participants included children with CCHD (unrepaired) and children with palliative shunts (e.g., Blalock-Taussig shunt, bidirectional Glenn shunt) within the age range of 2-18 years. Both outpatient and inpatient children with CCHD were enrolled. Only those patients who were willing to be part of the study were included. Children with known renal disease, kidney malformations, urinary tract abnormalities, those taking nephrotoxic drugs during the past three months (such as aminoglycosides), those who underwent cardiac catheterization within the past three months, and those who had undergone cardiac surgery (total correction) for CCHD were excluded from the study. The calculated sample size for the study was 94, which was derived by assuming the prevalence of CN in children with CCHD as 58.51%, alpha error of 0.05, and 95% confidence interval [8].

The renal impairment was measured with the impaired value of ACR >30 mg/g, spot urine protein-to-creatinine ratio (PCR) of >0.2 mg protein/mg creatinine, and estimated glomerular filtration rate (eGFR) of <90 mL/minute/1.73 m^2^. The eGFR was calculated using the creatinine-based "bedside Schwartz" equation (2009) for patients up to 18 years of age.

All eligible children, fulfilling the inclusion criteria, were enrolled after taking informed consent. Routine baseline investigations and clinical examination were done for all enrolled cases. Early morning urine samples were collected and analyzed for albumin, protein, and creatinine. Routine blood samples, including renal function tests, liver function tests, and complete blood count, were taken as part of the baseline investigations. Quantitative tests were done on a fully automated photometric analyzer, the Beckman Coulter AU 5800 instrument (Beckman Coulter, Inc., Brea, CA). Urine microalbumin levels were estimated by using an immunoturbidimetric test, and urinary protein levels were measured by using a photometric test using pyrogallol red.

The ACR, PCR, and eGFR were calculated, and individuals were labelled accordingly to estimate the prevalence of CN. The study population was further subgrouped based on age, type of pathology, HCT levels, and oxygen saturation levels; prevalence was calculated within these subgroups.

Statistical analysis

Microsoft Excel (Microsoft Corporation, Redmond, WA) and Statistical Package for the Social Sciences (version 26.0, IBM Corp., Armonk, NY) were used for statistical analysis. Continuous variables were summarized as means ± standard deviations for normal distribution data and as median (interquartile range) for nonparametric distribution data. Student's unpaired t-test was used to compare the means of continuous variables and normally distributed data. A value of p < 0.05 was considered statistically significant. Categorial comparison was made to see the differences in ACR, PCR, and eGFR between CHD subtypes using chi-square and Fisher's exact tests.

## Results

Ninety-eight children with CCHD were included in the study. The mean age was 6.33 ± 4.93 years, 55 were men (56.12%), 43 were women (43.87%), and the male:female ratio was 1.27:1. Distribution of CHD was as follows: double-outlet right ventricle with pulmonary stenosis (PS) (n = 10, 10.20%), TOF (n = 52, 53.06%), VSD with severe pulmonary arterial hypertension Eisenmenger's or severe PS (n = 9, 9.18%), TGA (n = 5, 5.10%), pulmonary atresia/tricuspid atresia (n = 6, 6.12%), and others (n = 16, 16.32%).

Out of the total 98 cases, 79 cases had albuminuria with an ACR of >30 mg/g of creatinine, which forms 80.61% of the study population. PCR was more than 0.2 mg protein/mg creatinine in 53 cases, forming 54.08% of the study population. eGFR was calculated by the bedside Schwartz formula. Thirty-nine cases had a GFR of less than 90 mL/minute, accounting for 39.79% of the study population. Two (n = 2) had nephrotic range proteinuria by urine PCR, and none of the patients were in CKD stage 4 or 5.

While analyzing the prevalence of renal impairment among different types of CHD, we found that tetralogy of Fallot (TOF) physiology had the highest prevalence, with 81% of TOF cases showing albuminuria greater than 30 mg/g of creatinine. Notably, all cases of transposition of the great arteries (TGAs) had significant albuminuria, indicating a prevalence of 100% in the TGA group. There were no significant differences in ACR levels between the different pathologies, suggesting that the specific type of cyanotic defect may not significantly affect the severity of renal injury. In terms of PCR, the highest prevalence was observed in the TGA group, with 80% showing significant proteinuria. However, there were no significant differences in glomerular filtration rate (eGFR) values between the different pathology groups. These findings demonstrate the varying prevalence of renal impairment across different CCHD pathologies, with TOF and TGA cases showing the highest percentages of renal dysfunction. The distribution of the above cases is shown in Table 1.

**Table 1:** Prevalence of renal impairment among different subtypes of cyanotic heart disease TOF: tetralogy of Fallot; VSD: ventricular septal defect; PAH: pulmonary arterial hypertension; PS: pulmonary stenosis; TGA: transposition of the great arteries; ACR: albumin-to-creatinine ratio; PCR: protein-to-creatinine ratio; eGFR: estimated glomerular filtration rate; DORV: double outlet right ventricle

Parameters	DORV/PS (n = 10)	TOF physiology (n = 52)	VSD with severe PAH (Eisenmenger)/PS (n = 9)	TGA (n = 5)	Pulmonary atresia/tricuspid atresia (n = 6)	Others (n = 16)	p value
ACR > 30 mg/g	9 (90%)	42 (81%)	7 (77%)	5 (100%)	5 (83%)	14 (87%)	0.4
PCR > 0.2 mg/mg	5 (50%)	24 (46%)	5 (55%)	4 (80%)	4 (66%)	12 (75%)	0.8
eGFR < 90 mL/minute	2 (20%)	24 (46%)	8 (88%)	2 (40%)	1 (16%)	6 (38%)	0.34

Subgroup analysis for CN was done based on age, HCT level, and oxygen saturation. Patients were divided into three groups on the basis of age (Table 2): 2-5, 6-10, and 11-18 years. The older age group (11-18 years) had a higher prevalence of renal impairment compared to the younger age groups. Specifically, the prevalence of albuminuria was found to be 66% in the 11-18-year age group, higher than the 55% prevalence in the two-to-five-year age group. Although age-wise differences in the proportion of children with ACR >30 mg/g were observed (55% in two to five years, 63% in 6-10 years, and 66% in 11-18 years), these differences were not statistically significant (chi-square test, p = 0.527). The apparent increase in prevalence with advancing age may reflect underlying disease progression or longer exposure to risk factors, but the absence of statistical significance suggests that the observed variation could be due to sample size limitations or random variation. Regarding proteinuria, significant protein excretion was observed in all age groups, but no significant differences were found between the different age groups. In terms of estimated glomerular filtration rate (eGFR), a higher prevalence of low eGFR was seen in the 11-18 year age group (57%) compared to the 6-10 year age group (42%) and the two-to-five-year age group (39%). The study revealed that among children with CCHD, the prevalence of renal dysfunction increased with age.

**Table 2:** Age-wise prevalence of renal impairment ACR: albumin-to-creatinine ratio; PCR: protein-to-creatinine ratio; eGFR: estimated glomerular filtration rate

Parameter	2-5 years (n = 58)	6-10 years (n = 19)	11-18 years (n = 21)	p value
ACR > 30 mg/g	32 (55%)	12 (63%)	14 (66%)	0.53
PCR > 0.2 mg/mg	28 (48%)	11 (58%)	13 (61%)	0.36
eGFR < 90 mL/minute	23 (39%)	8 (42%)	12 (57%)	0.72

Patients were analyzed for renal impairment in terms of HCT (Table 3) and oxygen saturation (Table 4). It was observed that the group with HCT levels greater than 65% had a higher prevalence of albuminuria, proteinuria, and lower eGFR. This led to the conclusion that elevated HCT levels (>65%) were associated with a higher prevalence of renal impairment, with ACR levels >30 mg/g in 92% (25/27) of patients in the high HCT group compared to 75% (53/71) of patients in the lower HCT group. The difference in ACR levels between the two groups was found to be statistically significant (p = 0.0001). Although the prevalence of proteinuria was higher in the group with higher HCT levels, no statistically significant difference was observed between the two groups based on PCR values. Similarly, there was no statistically significant difference in eGFR levels between the two groups. These findings suggest that elevated HCT levels may contribute to a higher prevalence of renal impairment in children with CCHD, emphasizing the importance of considering HCT as a potential risk factor in assessing renal health in these patients.

**Table 3:** Distribution of renal impairment cases based on HCT HCT: hematocrit; ACR: albumin-to-creatinine ratio; PCR: protein-to-creatinine ratio; eGFR: estimated glomerular filtration rate

Parameters	HCT ≤ 65% (n = 71)	HCT > 65% (n = 27)	p value
ACR ≥ 30 mg/g creatinine	53 (75%)	25 (92%)	0.0001
PCR ≥ 0.2 mg/mg creatinine	32 (45%)	20 (74%)	0.02
eGFR < 90	27 (38%)	11 (40%)	1.0

**Table 4:** Distribution of renal impairment cases based on SpO2 ACR: albumin-to-creatinine ratio; PCR: protein-to-creatinine ratio; eGFR: estimated glomerular filtration rate; SpO_2_: oxygen saturation

Parameters	SpO_2_ ≤ 70% (n = 44)	SpO_2_ > 70% (n = 54)	p value
ACR ≥ 30 mg/g creatinine	41 (93%)	38 (70%)	0.0001
PCR ≥ 0.2 mg/mg creatinine	28 (63%)	25 (46%)	0.67
eGFR < 90	10 (22%)	23 (42%)	0.29

The group with lower oxygen saturation (≤70%) had a higher prevalence of renal impairment, 93% (41/44), compared to the group with higher oxygen levels (>70%, prevalence of 70% (38/54). Significant differences were found in the ACR between the two groups, with the lower oxygen saturation group showing significantly higher ACR values (278 ± 157) compared to the higher oxygen saturation group (94 ± 100). However, no significant difference was observed in the urinary PCR levels between the groups. These findings suggest that lower oxygen saturation levels may contribute to an increased prevalence of renal impairment and more severe proteinuria in children with CCHD.

## Discussion

Although there are a few articles talking about the nephropathy occurring in congenital heart disease patients, there is still a paucity of data on the prevalence of nephropathy in cyanotic heart disease. Renal impairment is a well-recognized complication of CCHD and is often referred to as CN. The prevalence of CN varies from 30% to 50% in various studies [11-13].

Hongsawong et al. studied the prevalence of CN in a tertiary care hospital of Northern Thailand; the study population included children from one month to 15 years and reported the prevalence of CN to be 92.55% based on proteinuria staging and 58.51% on the basis of albuminuria staging [8]. Degree of hypoxia, elevated HCT, and long-standing disease are the risk factors for CN [14-17]. The pathophysiological interaction between the heart and kidney is known as cardiorenal syndrome (CRS) and is classified into five types (types I-V). CN is classified under type II CRS [18,19]. CN is associated with the development of chronic kidney disease later in life. Dimopoulos et al. found higher mortality in adult congenital heart disease patients with moderate to severe reduced GFR [2].

In our study, we used serum creatinine, urine albumin, urine protein, and urine creatinine to calculate ACR, PCR, and eGFR to diagnose CN. Out of the total 98 cases, 79 cases had albuminuria with an ACR of >30 mg/g of creatinine, which forms 80.61% of the study population. PCR was more than 0.2 mg/mg of creatinine in 53 cases, forming 54.08% of the study population. eGFR was calculated by the bedside Schwartz formula; 39 cases had an eGFR of less than 90 mL/minute, forming 39.79% of the study population. As previous studies have shown, microalbuminuria is an early and reliable marker of glomerular damage. Taking microalbuminuria as the reference, we found that the prevalence of renal impairment was 80.61% among the CCHD patients. The prevalence of CN in our study is in tune with 92.55%, as found by Hongsawong et al. in their study [8].

On the basis of age, we categorized them into three major groups: 2-5, 6-10, and 11-18 years of age. The prevalence of CN in the 11-18-year age group was higher than that in the two-five-year age group (66% vs. 55%), which indicates that long-standing uncorrected CCHD causes progressive renal dysfunction. Although age-wise differences in the proportion of children with ACR >30 mg/g were observed (55% in two to five years, 63% in 6-10 years, and 66% in 11-18 years), these differences were not statistically significant (chi-square test, p = 0.527). The higher prevalence of CN in older children and adults is in accordance with Dittrich et al., Agras et al., and Krull et al., which showed that increasing age is a risk factor for CN [6,9,16].

Categorizing them based on different pathologies gave us no significant difference in the ACR, PCR, and eGFR levels, suggesting that the prevalence of renal impairment is not affected by the type of CCHD. In contrast to our findings, previous studies have shown a significantly higher prevalence of CN in Eisenmenger and Fontan patients [20].

However, we got significant results when we compared the groups based on HCT levels. We divided our study population into groups having an HCT level of less than or equal to 65% or more than 65%. Raised HCT (>65%) was associated with a higher prevalence of renal impairment (high ACR: 92% vs. 75%) compared to the lower HCT level group. The ACR was significantly higher in the higher HCT group, with a p value of 0.0001. Studies conducted in the past showed that a higher degree of polycythemia had a linear relation with proteinuria. There are several mechanisms postulated for the hyperviscosity-related glomerular injury [20,21]. The hyperviscosity-associated fall in peritubular blood flow often causes the glomerular capillary pressure to rise, which further results in significant proteinuria [22]. It has also been postulated that polycythemia results in increased viscosity of the ultrafiltrate, which in turn leads to release of NO, causing vasodilatation, which further complicates as glomerulosclerosis and glomerulomegaly [23]. From this, we can postulate that the basic etiopathogenesis behind renal impairment is polycythemia and the resultant hyperviscosity, which causes glomerular damage leading to significant proteinuria. Repeated phlebotomies are done to decrease the HCT in these patients. It was seen that a fall in HCT after phlebotomy was associated with a fall in proteinuria and an eventual rise in GFR, suggesting that hyperviscosity might be playing a critical role in the high-pressure gradient between the afferent and efferent arterioles, which fell significantly after phlebotomy [1,3].

Hypoxemia, lower oxygen saturation levels, was also found to be associated with increased albuminuria, giving us a clue that hypoxemia is a significant contributor to the pathogenesis of renal glomerular and tubular damage.

With the study conducted in a tertiary care hospital in India, despite its limitations, including being a single-center study, analyzing a single set of samples, and lacking long-term follow-up, it successfully calculated the prevalence of CN and its associations. Using microalbuminuria as a reliable defining parameter, we concluded that 80.61% of all the CCHD patients have latent nephropathy, which may be unmasked later in life. We also found that the prevalence of albuminuria was higher in patients with higher HCT levels, supporting that the pathogenesis of renal injury is associated with hyperviscosity. Currently, the term CN is not that familiar, so we usually miss this complication occurring in cyanotic children. This may be due to a lack of knowledge and insufficient data supporting the evidence. However, proteinuria should be addressed promptly, as it can lead to complete renal shutdown in the long term. Therefore, we need to be vigilant regarding this treatable complication. Watanabe et al. in their study showed that ACE inhibitors were somewhat effective in decreasing the proteinuria, although repeated phlebotomies were also considered in some studies [24]. Both of these treatment options are only temporary, with surgical repair being the only permanent solution. Therefore, with early recognition of CN, along with regular monitoring of proteinuria, GFR, and hypertension, renal injury can be prevented, leading to further improvement in overall disease progression.

## Conclusions

This study reveals a high prevalence of latent nephropathy among children with CCHD. Older age, elevated haematocrit levels, and hypoxemia were strongly associated with renal dysfunction. Early recognition and monitoring of Impaired renal function are crucial, with surgical intervention being the definitive treatment, while ACE inhibitors and phlebotomy offer temporary management options.
